# Comparison of the efficacy and safety of metallic ureteral stent versus polymer ureteral stent for patients with malignant ureteral obstruction: a meta-analysis of comparative trials

**DOI:** 10.1007/s00345-026-06287-3

**Published:** 2026-02-24

**Authors:** Jung Hoon Kim, Yun-Jung Yang, Joongwon Choi, Yong Seong Lee, Kyungchan Min, Jong Hyun Tae, Se Young Choi, Eun-Jung Yang, Chung Un Lee

**Affiliations:** 1 Department of Urology, Chung-Ang University Gwangmyeong Hospital, Chung-Ang University College of Medicine, Gwangmyeong, Gyeonggi-Do Republic of Korea; 2 Department of Convergence Science, College of Medicine, Catholic Kwandong University International St. Mary’s Hospital, Incheon, Korea; 3 Department of Urology, Chung-Ang University Hospital, Chung-Ang University College of Medicine, Seoul, South Korea; 4 Department of Plastic and Reconstructive Surgery, Institute for Human Tissue Restoration, Yonsei University College of Medicine, Seoul, Korea

**Keywords:** Malignant ureteral obstruction, Metallic ureteral stent, Polymer ureteral stent, Meta-analysis

## Abstract

**Purpose:**

This study aimed to evaluate and compare the clinical performance of metallic and polymer ureteral stents in patients with malignant ureteral obstruction (MUO), with a focus on complications and success rates.

**Methods:**

We conducted a meta-analysis of seven studies, obtained by searching various databases from inception to February 2025, involving patients with MUO treated with metallic or polymer stents. Study quality was assessed using the RoB 2 tool for randomized controlled trials and the ROBINS-I tool for non-randomized studies. Primary outcomes included success rates at 1, 3, 6, and 12 months, and complication rates. Odds ratios (ORs) with 95% confidence intervals (CIs) were calculated using a random-effects model. When data were available, exploratory analyses were performed according to metallic stent type.

**Results:**

The success rates at 3, 6, and 12 months were consistently higher in patients with metal stents (ORs 5.14, 2.70, and 3.67, respectively) than in those with polymer stents. The overall rate of complications did not differ significantly between the two groups; however, a higher risk of complications was observed in studies using covered metallic stents (OR 4.45, 95% CI 1.57–12.62).

**Conclusions:**

Compared with polymer ureteral stents, metallic ureteral stents demonstrated higher success rates in patients with MUO, particularly at longer follow-up, albeit with an increased risk of complications. These findings suggest that metallic stents may represent a favorable option in carefully selected patients.

**Supplementary Information:**

The online version contains supplementary material available at 10.1007/s00345-026-06287-3.

## Introduction

Malignant ureteral obstruction (MUO) may arise from direct tumor invasion, lymphatic spread, or external compression by metastatic lesions. If left untreated, MUO can progress to hydronephrosis, renal dysfunction, or potentially life-threatening urosepsis. Notably, > 50% patients with MUO experience at least one complication despite appropriate management, which can be particularly detrimental given their limited median survival of 5–6.8 months [1, 2].

Therefore, timely urinary diversion plays a crucial role in preserving renal function, relieving symptoms, and facilitating the continuation of systemic oncological treatments. The two primary decompression methods are percutaneous nephrostomy and retrograde ureteral stent insertion. Percutaneous nephrostomy is effective; however, its external drainage system causes discomfort and impairs quality of life [3]. Consequently, polymer double-J stents are commonly used as internal drainage alternatives, particularly in palliative care, owing to their technical simplicity and wide availability [4]. However, these stents are prone to complications such as encrustation, obstruction, and infection and require frequent replacements, typically every 3–6 months [5, 6].

Consequently, various types of metallic ureteral stents that provide superior durability and resistance to external compression have been developed. Stents such as Resonance, Urexel, Uventa, and Memokath have demonstrated encouraging outcomes in clinical practice. and reportedly provide prolonged patency and reduce the frequency of interventions, particularly in patients requiring extended periods of urinary diversion [7–10]. Nevertheless, metallic stents are not without drawbacks; complications such as hematuria, migration, and technical difficulties during placement have been reported [11]. Additionally, while some studies have demonstrated the clear advantages of metallic stents, others have reported no significant differences or suggested higher complication rates. Variations in patient populations, tumor types, stent designs, and outcome definitions likely contributed to the heterogeneity of the results [12–14], underscoring the need for systematic and comprehensive evaluation of the relative clinical performance of each stent type.

Therefore, we conducted a meta-analysis to compare the clinical outcomes, including patency and complications, of metallic and polymer ureteral stents in patients with MUO by synthesizing data from a broad range of studies.

## Materials and methods

The study protocol was registered in PROSPERO (registration number: CRD420251060566), and the meta-analysis was performed according to the guidelines outlined in the Preferred Reporting Items for Systematic Reviews and Meta-Analyses 2020 (PRISMA 2020) statement.

### Search strategy and study selection

A proficient medical librarian performed electronic searches of the Web of Science, Embase, Cochrane Central Register of Controlled Trials, Scopus, and PubMed databases (via the National Library of Medicine) from inception to February 2025 (Supplementary Table 1). The following search terms were used: polymer stent, polymer ureteral stent, double-J stent, double J stent, polyurethane stent, metal stent, metallic stent, metallic ureteral stent, covered metal stent, covered metallic stent, metals, malignant ureteral obstruction, and malignant ureteric obstruction. Comparator terms such as versus, comparison, or compare were intentionally excluded to avoid narrowing the scope of the search. The reference lists of all included articles and related reviews were manually reviewed for additional eligible research that might have been missed by the electronic search. We also used the International Clinical Trials Registry Platform and ClinicalTrials.gov to identify ongoing trials.

After eliminating duplicates, all references were imported into Covidence (www.covidence.org). Two members (J. C. and Y. J. Y.) of the study team independently assessed each reference and abstract based on the predetermined selection criteria. In case of disagreement between the two members, a majority decision was reached based on the opinion of a third team member (J. H. K. or C. U. L.). Abstracts were screened to exclude studies that did not meet the inclusion criteria. Two members of the study team independently conducted full-text reviews of the included studies.

### Inclusion and exclusion criteria

Predefined inclusion and exclusion criteria were established before initiating the search. Comparative trials meeting the following criteria were included: studies that (1) compared polymer ureteral stents and metallic ureteral stents; (2) enrolled patients with ureteral obstruction secondary to malignancy preoperatively; and (3) provided at least one indicator for analysis, such as success rate or complications. Studies that met the following criteria were excluded: (1) clinical trials that did not compare procedures between polymer ureteral stents and metallic ureteral stents; (2) studies without available full text (including reviews, conference abstracts, editorial notes/letters, and narrative reviews); (3) studies lacking parameters for comparative analysis of the results; and (4) studies that were not published in English.

### Data extraction

General information, such as the first author's name, year when the study was performed, country where the study was performed, study type, and outcomes, was extracted from the included studies. The extracted outcomes included postoperative success rates at 1, 3, 6, and 12 months, along with any complications.

Additionally, data on the number of participants, study design, type of metallic stent (covered or full-length), follow-up duration, and the definition of “success” were collected to describe study-level baseline characteristics. Two reviewers independently extracted all data, and discrepancies were resolved through discussion and consensus.

### Quality assessment

The risk of bias of the included studies was assessed according to study design. Randomized controlled trials (RCT) were evaluated using the Cochrane Risk of Bias 2 (RoB 2) tool, which assesses bias arising from the randomization process, deviations from intended interventions, missing outcome data, outcome measurement, and selective reporting [15].

Non-randomized comparative studies were assessed using the Risk Of Bias In Non-randomized Studies of Interventions (ROBINS-I) tool, which evaluates bias due to confounding, selection of participants, classification of interventions, deviations from intended interventions, missing data, measurement of outcomes, and selective reporting [16].

Two reviewers independently performed the risk-of-bias assessment. Disagreements were resolved through discussion or consultation with a third reviewer.

### Statistical analysis

The pooled risk of complications and success rates at 1, 3, 6, and 12 months were estimated for patients diagnosed with MUO and treated with either metal or polymer stents. In studies that reported only proportions (%), the number of events was calculated by multiplying the proportion with the total number of participants [17]. The odds ratios (ORs) and 95% confidence intervals (CIs) were calculated using a random-effects model with the restricted maximum likelihood method. When data were available, exploratory analyses were performed according to metal stent type (full-length vs covered). Heterogeneity across studies was assessed using the I^2^ statistic and the Chi-square-based Q-test. Substantial heterogeneity was defined as an I^2^ value of > 50% combined with p < 0.1, while low heterogeneity was defined as an I^2^ value of < 50% and p > 0.1. Clinical and methodological heterogeneity was anticipated due to differences in patient populations, malignancy types, stent characteristics, and definitions of clinical success across the included studies. Given the scarcity of available studies, results from RCTs and nonrandomized studies were not analyzed separately due to the limited number of randomized trials. Statistical significance was set at p < 0.05. All statistical analyses were performed using the STATA/MP v18 (StataCorp, College Station, TX, USA) software. Publication bias was not formally assessed using funnel plots or Egger’s test because the number of included studies for each outcome was fewer than ten.

## Results

### Study characteristics

**Fig. 1:**
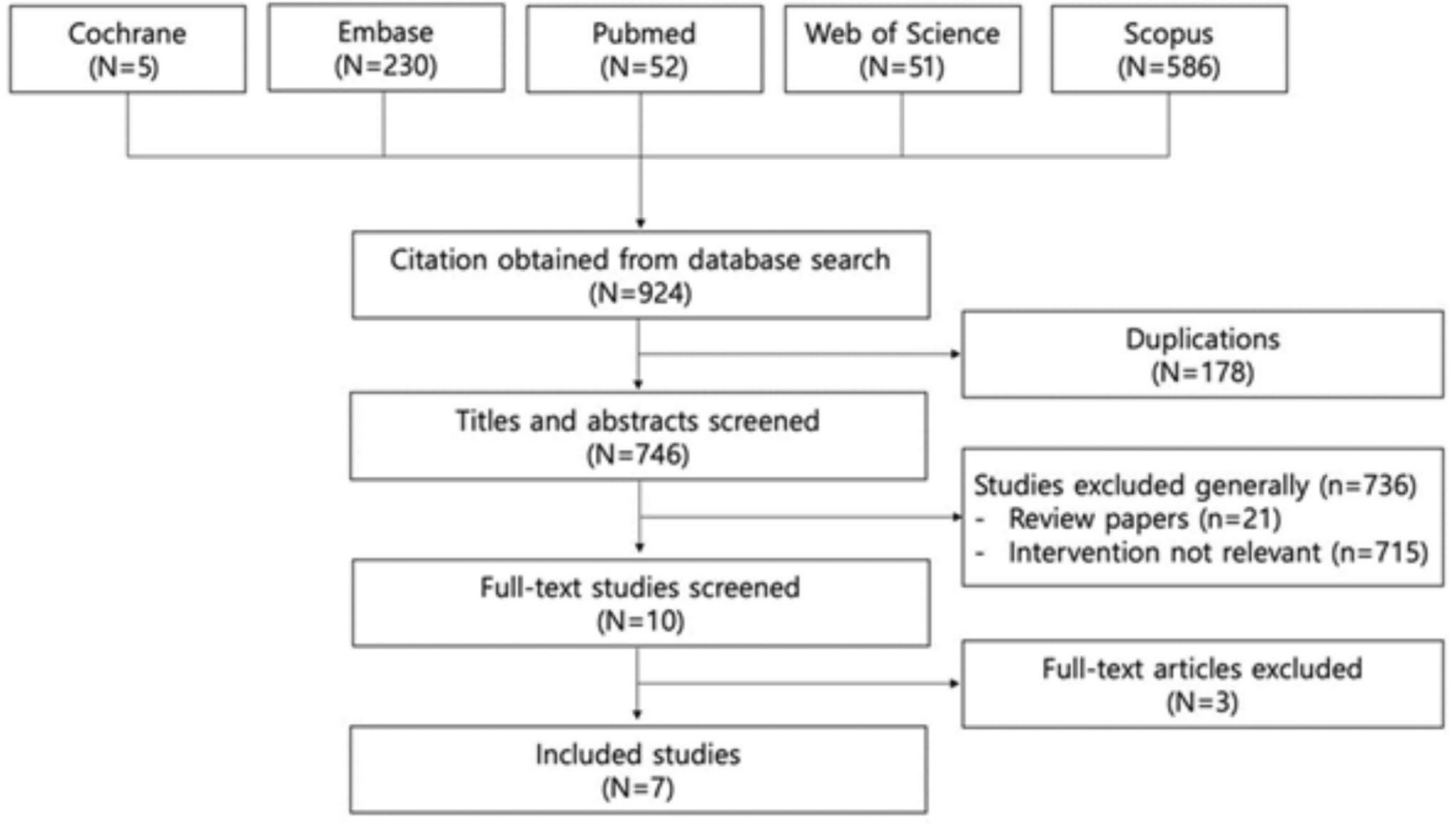
Flow diagram of studies identified, included, and excluded from the analysis

As shown in Fig. 1, 924 articles were preliminarily retrieved through the electronic search, of which seven studies were finally included. The characteristics of the enrolled studies are summarized in Table 1, which includes two RCTs and five non-RCT studies, with 247 and 235 patients in the metallic and polymer ureteral stent groups, respectively.

**Table 1:** Summary of meta-analysis on complication and success rates between metal and polymer stents in patients with malignant ureteral obstruction. (Subgroup results based on a single study are presented for descriptive purposes only.)

Outcome measures	Studies (n)	Metal stent		Polymer stent		OR (95% CI)	Heterogeneity				Refs.
		Total (n)	Event (n)	Total (n)	Event (n)		df	Q	P_-hetero_	I^2^ (%)	
*(A) Overall*											
Success rate											
1-month	3	109	99	115	91	3.79 (0.74–19.40)	2	4.04	0.132	50.79	Chung et al. [18], Han et al. [19]
3-month	4	119	105	124	83	5.14 (1.81–16.45)	3	4.86	0.182	40.72	Chung et al. [18], Han et al. [19], Kim et al. [20, 21]
6-month	5	147	118	161	102	2.70 (1.52–4.79)	4	2.11	0.716	0.00	Chen et al. [10], Chung et al. [18], Han et al. [19], Kim et al. [20, 21]
12-month	5	162	119	152	68	3.67 (1.76–7.62)	4	7.08	0.131	44.13	Asakawa et al. [12], Chen et al. [10], Chung et al. [18], Kim et al. [20, 21]
Complication	5	198	58	154	41	1.55 (0.50–4.79)	4	12.18	0.016	68.76	Asakawa et al. [12], Chen et al. [10], Kim et al. [20, 21], Ohtaka et al. [23]
*(B) Full-length metal stent*											
Success rate											
6-month*	1	28	28	37	31	11.76 (0.63–218.24)	–	–	–	–	Chen et al. [10]
12-month	2	81	67	65	33	5.49 (0.81–37.19)	1	4.26	0.039	76.51	Asakawa et al. [12], Chen et al. [10]
Complication	3	134	31	115	36	0.69 (0.36–1.30)	2	3.17	0.205	0.00	Asakawa et al. [12], Chen et al. [10], Ohtaka et al. [23]
*(C) Covered metal stent*											
Success rate											
1-month	3	109	99	115	91	3.79 (0.74–19.40)	2	4.04	0.132	50.79	Chung et al. [18], Han et al. [19]
3-month	4	119	105	124	83	5.14 (1.81–16.45)	3	4.86	0.182	40.72	Chung et al. [18], Han et al. [19], Kim et al. [20, 21]
6-month	4	119	90	124	71	2.55 (1.42–4.56)	3	1.09	0.779	0.00	Chung et al. [18], Han et al. [19], Kim et al. [20, 21]
12-month	3	81	52	87	35	3.13 (1.29–7.57)	2	2.65	0.266	33.24	Chung et al. [18], Kim et al. [20, 21]
Complication	2	64	27	39	5	4.45 (1.57–12.62)	1	0.06	0.807	0.00	Kim et al. [20, 21]

*Results based on a single study; heterogeneity statistics not applicable

### Risk of bias assessment

Of the seven included studies, two were RCT trials and five were non-randomized comparative studies. The randomized controlled trials were assessed using the RoB 2 tool. One trial was judged to have low risk of bias across all domains, while the other showed some concerns, mainly related to the randomization process and deviations from intended interventions. No randomized trial was judged to be at high risk of bias. The non-randomized comparative studies were evaluated using the ROBINS-I tool. Overall, most studies were judged to have a moderate risk of bias, primarily due to potential confounding and patient selection inherent to the retrospective study design. One study was assessed as having a serious risk of bias, mainly related to confounding. No study was judged to be at critical risk of bias. A detailed domain-based risk-of-bias assessment for each study is provided in Supplementary Table 2.

### Success rate

We performed a comparative evaluation of the success rates of metallic and polymer stents at 1, 3, 6, and 12 months. A summary of the meta-analysis for the success rates in patients with malignant ureteral obstruction is presented in Table 1.

#### 1-month success rate

**Fig. 2:**
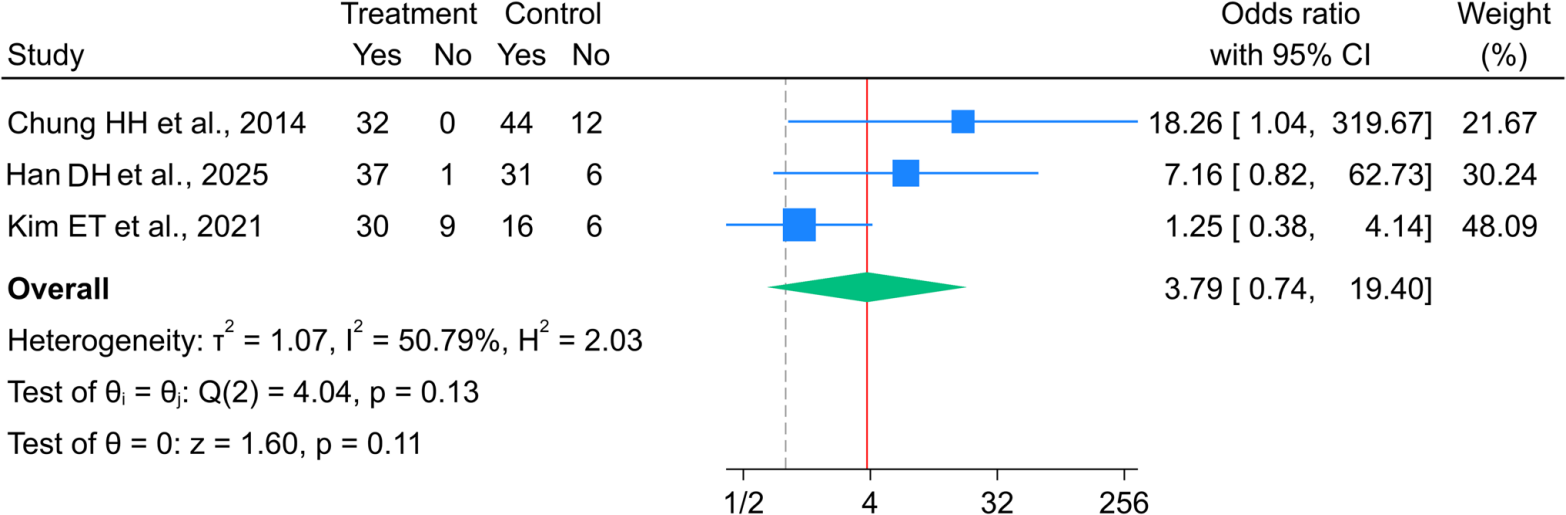
Forest plot for 1-month success rate between metal and polymer stent groups. *The vertical reference line indicates no effect (odds ratio = 1), and the diamond represents the overall pooled effect estimate

Three studies [18–20] comprising 109 and 115 patients in the metallic and polymer stent groups, respectively, were included in the analysis of the 1-month success rate. A subgroup analysis based on stent design was not performed because all studies included in this comparison utilized covered metal stents. The overall OR (95% CI) for the 1-month success rate between the metal and polymer stent groups was 3.79 (0.74–19.40 (Fig. 2)). Although the individual study variance included moderate heterogeneity (I^2^ = 50.8%), it was not statistically significant (p = 0.132).

#### 3-month success rate

**Fig. 3:**
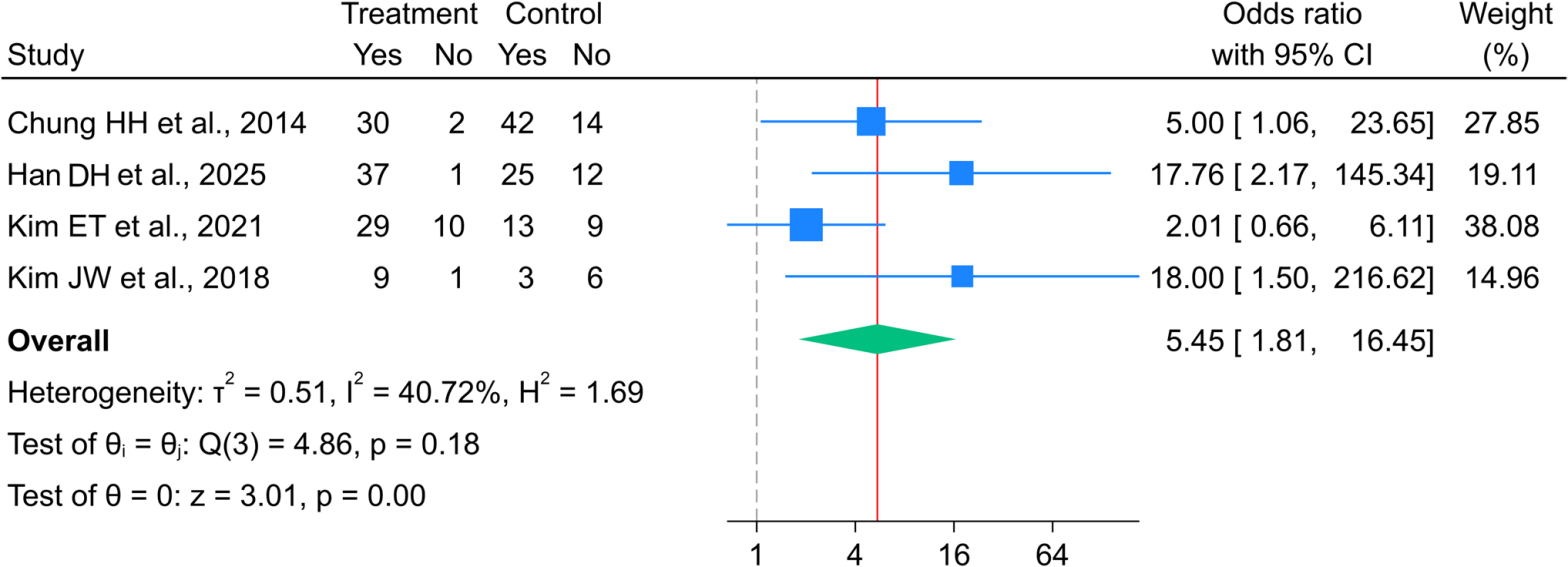
Forest plot for 3-month success rate between metal and polymer stent groups. *The vertical reference line indicates no effect (odds ratio = 1), and the diamond represents the overall pooled effect estimate

Four studies [18–21] involving 119 patients with metal stents and 124 with polymer stents reported 3-month success rates. Similar to the 1-month success rate data, only covered metal stents were used in the studies reporting 3-month success rates; therefore, a subgroup analysis could not be performed. The pooled 3-month success rate in the covered metal stent group was 5.14 times higher than that in the polymer stent group (95% CI 1.81–16.45), with low heterogeneity (I^2^ = 40.72%, p = 0.182 (Fig. 3)).

#### 6-month success rate

**Fig. 4:**
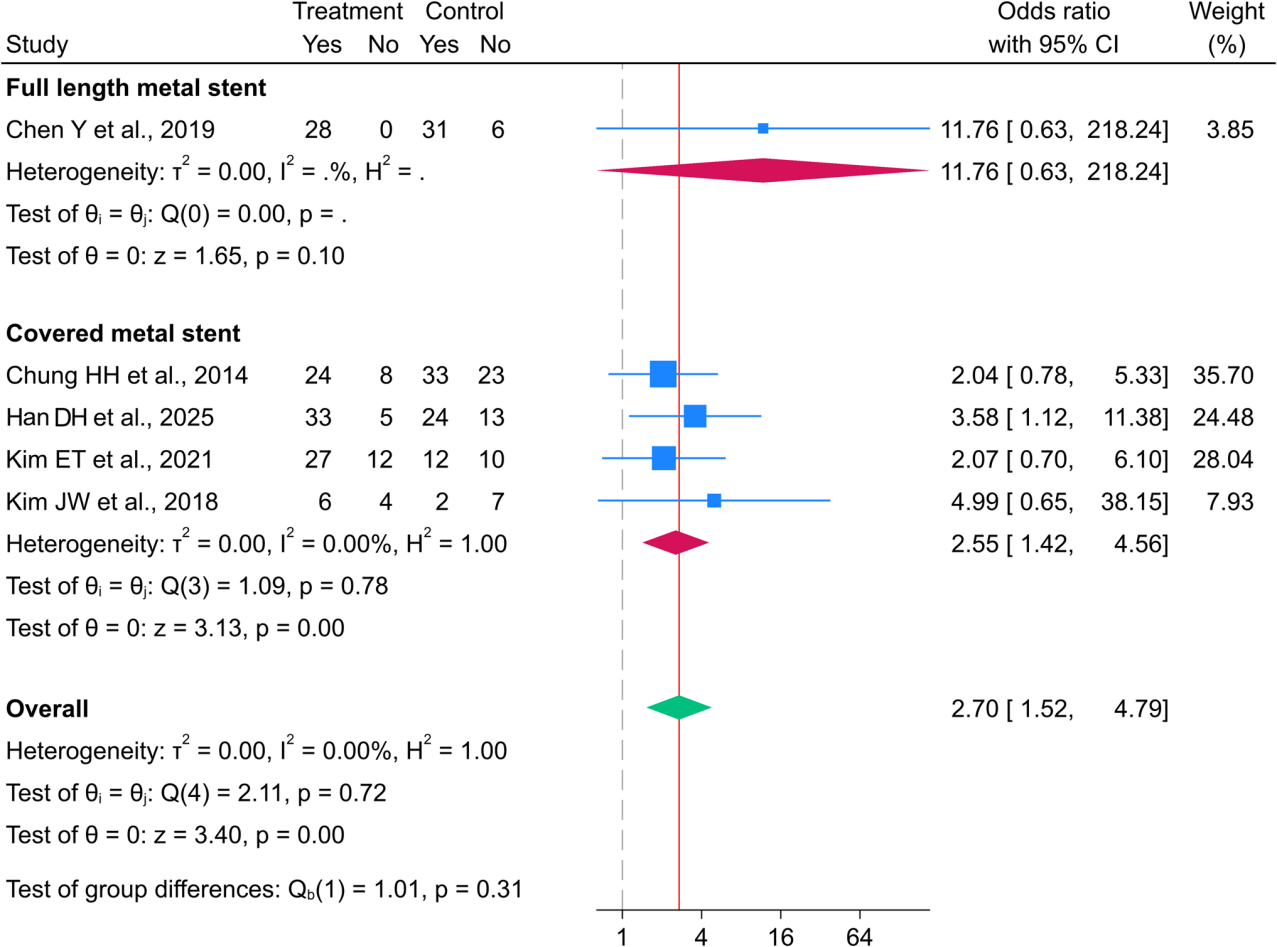
Forest plot for 6-month success rate between metal and polymer stent groups. *The vertical reference line indicates no effect (odds ratio = 1), and the diamond represents the overall pooled effect estimate

Five studies, including 147 and 161 patients in the metal and polymer stent groups, respectively, assessed the 6-month success rate [10, 18–21]. The overall 6-month success rate was significantly higher in the metal stent group than that in the polymer stent group (OR 2.70; 95% CI 1.52–4.79), with low heterogeneity (I^2^ = 0.00%, p = 0.716 (Fig. 4)). The OR for the 6-month success rate in the full-length metal stent group was 11.76 times higher than that in the polymer stent group (95% CI 0.63–218.27 (Fig. 4)). The pooled 6-month success rate in the covered metal stent group was significantly increased compared to the polymer stent group (OR 2.55; 95% CI 1.42–4.56), with low heterogeneity (I^2^ = 0.00%, p = 0.779 (Fig. 4)).

#### 12-month success rate

**Fig. 5:**
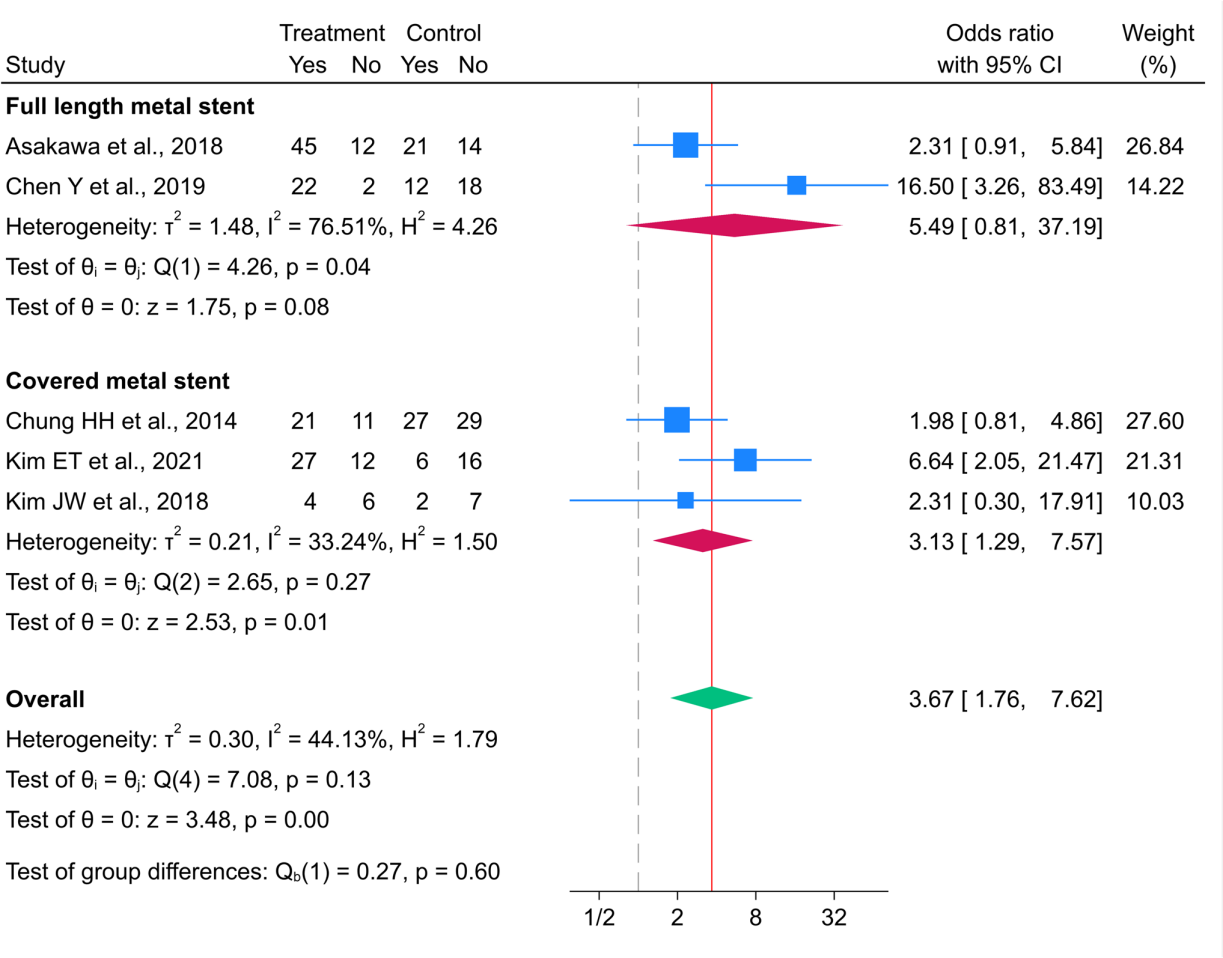
Forest plot for 12-month success rate between metal and polymer stent groups. *The vertical reference line indicates no effect (odds ratio = 1), and the diamond represents the overall pooled effect estimate

Five studies, [10, 18, 20–22] including 162 and 152 patients in the metal and polymer stent groups, respectively, reported 12-month success rates. The overall success rate in the metal stent group was significantly higher than that in the polymer stent group (OR 3.67; 95% CI 1.76–7.62), with low heterogeneity (I^2^ = 44.13%, p = 0.131 (Fig. 5)). After stratification by metal stent type, the pooled OR in the full-length metal stent subgroup was 5.49 (95% CI 0.81–37.19) compared to the polymer stent group, with substantial heterogeneity (I^2^ = 76.51%, p = 0.039 (Fig. 5)). Meanwhile, the overall 12-month success rate for the covered metal stent group was significantly higher than that for the polymer stent group (OR 3.13; 95% CI 1.29–7.57), with low heterogeneity (I^2^ = 33.24%, p = 0.266 [Fig. 5]).

### Complications

**Fig. 6:**
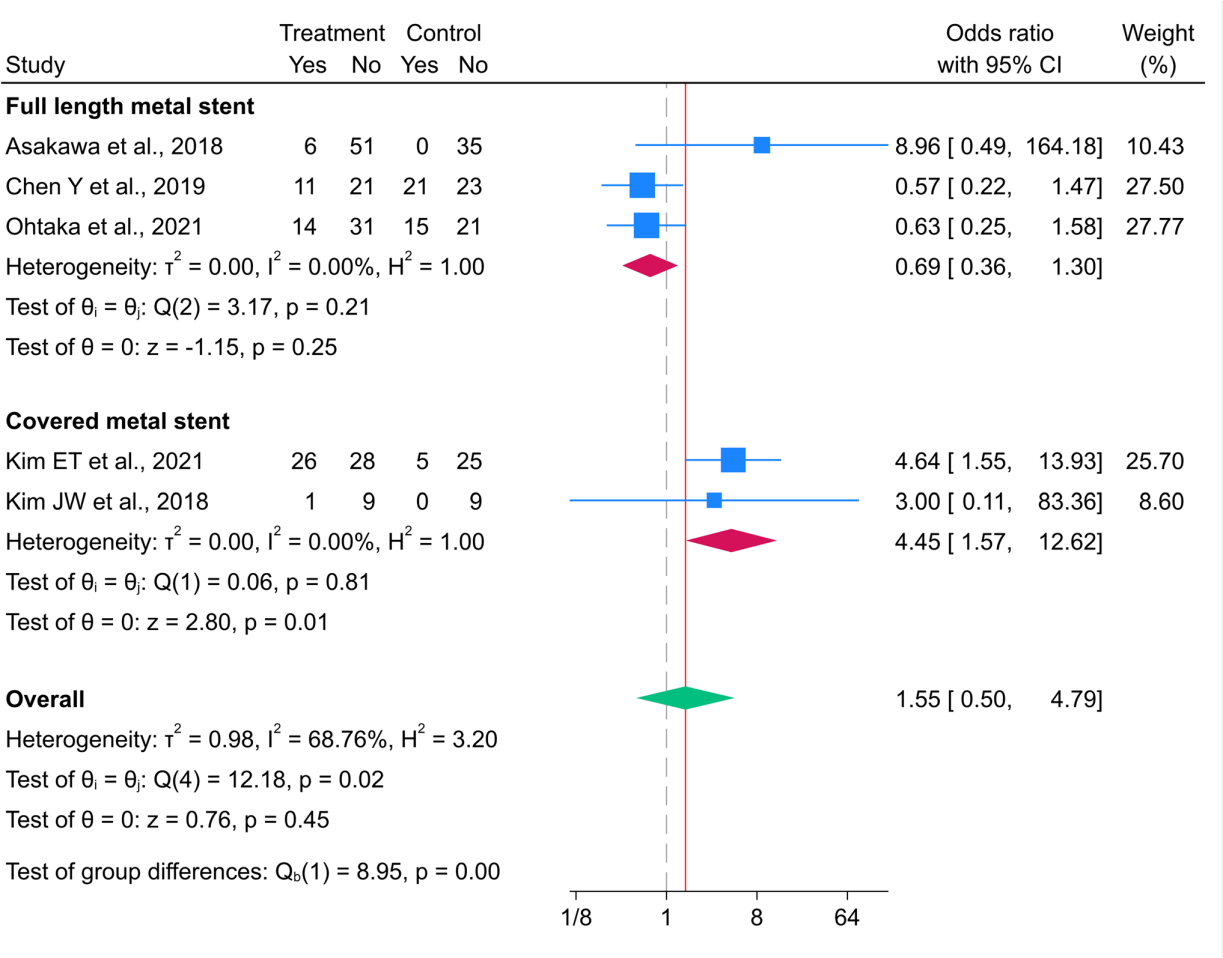
Forest plot for the incidence of complications between metal and polymer stent groups. *The vertical reference line indicates no effect (odds ratio = 1), and the diamond represents the overall pooled effect estimate

The five studies used to assess complications with stent insertion included 198 and 154 patients in the metal and polymer stent groups, respectively [10, 20–23]. The overall incidence of complications was not significantly different between the groups (OR 1.55 (95% CI 0.50–4.79), with substantial heterogeneity (I^2^ = 68.76%, p = 0.016 (Fig. 6)). The pooled rate of complications in studies using full-length metal stents was not significantly different between metal and polymer stent groups (OR 0.69, 95% CI 0.36–1.36), with low heterogeneity (Fig. 6). In contrast, the pooled OR (95% CI) for the incidence of complications in the covered metal stent group compared to that in the polymer stent group was 4.45 (1.57–12.62), indicating a significantly higher risk of complications, with low heterogeneity (I^2^ = 0.00%, p = 0.807 (Fig. 6)). Table 1 summarizes a meta-analysis of the complications of metal and polymer stents in patients with MUO.

## Discussion

In this meta-analysis, we provided a comprehensive comparison of metallic and polymer ureteral stents for the management of MUO. Our findings demonstrate that metallic stents are associated with higher stent patency at 3, 6, and 12 months compared to polymer stents, despite a higher incidence of complications. These results align with those of previous studies, suggesting that metallic stents offer enhanced resistance to extrinsic tumor compression and reduce the need for frequent re-intervention in appropriately selected patients [4, 6, 7, 10].

Although polymer double-J stents are widely used for internal urinary drainage, particularly in palliative settings, they are frequently associated with various complications. Consequently, a variety of metallic ureteral stents have been developed with the aim of improving durability and resistance to external compression. Covered metallic stents, such as UVENTA, Allium and UREXEL, are designed to prevent tumor ingrowth through polymer encapsulation of the metal mesh, thereby maintaining long-term patency. However, this mechanical advantage comes with a tradeoff of higher complication rates, including hematuria, stent migration, and patient discomfort, likely due to their stronger radial force [3, 9, 24]. Contrastingly, uncovered full-length stents, like the Resonance^®^ stent, offer a more favorable safety profile but may be less effective in patients with aggressive local invasion [5, 22]. Meanwhile, segmental thermoexpandable stents, such as Memokath 051, have shown variable performance, with some reports indicating high failure or migration rates in long or complex strictures [8, 11].

Beyond device design, the two stent materials serve fundamentally different clinical purposes. Because polymer ureteral stents are not designed for long-term indwelling, the higher patency observed with metallic stents at longer follow-up intervals may reflect differences in intended device lifespan rather than intrinsic superiority. Polymer double-J stents are intended for short-term use and typically require scheduled replacement every 3–6 months, whereas metallic stents are engineered for long-term indwelling to minimize the need for repeated procedures. Consequently, direct comparisons of outcomes at 6 or 12 months may not indicate true clinical equivalence but rather reflect two distinct management strategies. In practice, metallic stents may be advantageous for patients with longer expected survival or those needing durable urinary drainage, while polymer stents remain appropriate for short-term or palliative settings.

Stent selection must be tailored to the patient’s overall condition, anatomical characteristics, and therapeutic goals. Accordingly, several prognostic factors such as bladder invasion, bilateral obstruction, presence of severe hydronephrosis, and baseline renal function have been shown to influence outcomes irrespective of the stent type [14, 25, 26]. Moreover, the ability to receive systemic oncologic treatment following urinary diversion may be a more critical determinant of survival than the stent choice itself, underscoring the importance of holistic clinical evaluation [13, 27]. Furthermore, recent advancements in stent technology have supported the evolving role of metallic stents. Qing et al. reported encouraging results with an allium-covered metal stent, noting good mid-term patency and low migration risk, especially with close follow-up in patients with bulky pelvic disease [28]. Similarly, Lee et al. described favorable outcomes using a novel silicone-covered metallic mesh stent, including stable indwelling duration and improved renal function [29]. Miyazaki et al. demonstrated the durability of the Resonance® stent even in anatomically complex cases, with minimal encrustation and obstruction [30]. Collectively, these data reflect ongoing innovations that may mitigate complications associated with earlier-generation stents.

Notably, the definition of “stent success” varied considerably among the included studies. Some authors defined success by improvement in renal function or serum creatinine, whereas others used radiologic patency or the absence of reintervention as endpoints. Because these heterogeneous criteria were adopted as reported without harmonization, the pooled estimates in this analysis represent overall trends rather than precise quantitative measures. This variation underscores the need for standardized outcome definitions.

An exploratory analysis according to metallic stent design was performed to reflect real-world diversity in stent characteristics; however, these findings should be interpreted cautiously due to limited study numbers. Additionally, the inclusion of patients with heterogeneous malignancies and various treatment settings improved the external validity of our findings. Thus, our study provides actionable data to inform stent selection in palliative care by focusing on endpoints, such as patency, complications, and reintervention rates.

Nonetheless, this study has several limitations. First, although randomized controlled trials were included, the overall body of evidence was derived predominantly from retrospective cohort studies. As a result, the strength of the conclusions is largely influenced by the limitations inherent to non-randomized designs. According to the ROBINS-I assessment, these studies generally exhibited a moderate risk of bias, primarily due to factors such as selection bias, residual confounding, and incomplete outcome reporting. Moreover, the limited number of randomized controlled trials precluded separate analyses according to study design, and the pooled results should therefore be interpreted in the context of differing levels of evidence. Second, formal assessment of publication bias was not feasible because of the small number of included studies. Complications were also analyzed as a pooled outcome, as individual adverse events were inconsistently reported across studies, which precluded stratified analyses by specific complication types. Third, clinical heterogeneity across the included studies—including variations in malignancy type, prior treatments (e.g., radiotherapy), stent insertion techniques (retrograde vs. antegrade), and definitions of clinical success—may have limited the generalizability of the findings [3, 4, 14, 31]. Finally, the lack of patient-level data prevented subgroup analyses based on detailed clinical characteristics, such as tumor histology, ECOG performance status, and baseline renal function.

## Conclusions

Compared with polymer ureteral stents, metallic ureteral stents demonstrated higher success rates in patients with MUO, particularly at longer follow-up intervals, albeit with an increased risk of complications. These findings suggest that metallic stents may represent a reasonable option in carefully selected patients. However, differences observed at longer follow-up should be interpreted in the context of differing intended indwelling durations between stent types, rather than as evidence of intrinsic device superiority. Further well-designed prospective studies are warranted to better define optimal stent selection strategies in this population.

## Supplementary Information

Below is the link to the electronic supplementary material.

Supplementary file1 (DOCX 21 kb)

## Data Availability

The data supporting the findings of this study are available upon reasonable request (contact the CUL).

## Abbreviations

CI: Confidence interval
MUO: Malignant ureteral obstruction
OR: Odds ratio
PRISMA 2020: Reporting Items for Systematic Reviews and Meta-Analyses 2020
RCT: Randomized controlled trials
RoB 2: Cochrane Risk of Bias 2
ROBINS-I: Risk of Bias In Non-randomized Studies of Interventions
