# Supplementary material for: Comparison of the efficacy and safety of metallic ureteral stent versus polymer ureteral stent for patients with malignant ureteral obstruction: a meta-analysis of comparative trials
Source: World J Urol. 2026 Feb 24;44(1):192. doi: 10.1007/s00345-026-06287-3 (PMC12932312; doi:10.1007/s00345-026-06287-3)
Supplement: Supplementary file 1 — Supplementary file1 (DOCX 21 kb) [file 345_2026_6287_MOESM1_ESM.docx]

**Supplement Table 1. Search strategy for systemic review**

1) PubMed

| No. | Query | Results (n) |
| --- | --- | --- |
| 1 | “malignant ureteral obstruction” OR “malignant ureteric obstruction” OR "ureteral obstruction"[Mesh] AND "neoplasms"[MeSH]) | 3,157 |
| 2 | “metal stent” OR “metallic stent” OR “metallic ureteral stent” OR “covered metal stent” OR “covered metallic stent” OR "metals"[MeSH] | 1,290,661 |
| 3 | “polymer stent” OR “polymer ureteral stent” OR “double-J stent” OR “double J stent” OR “polyurethane stent” OR “polymers”[MeSH] | 1,017,426 |
| 4 | #1 AND #2 AND #3 | 52 |

2) Embase

| No. | Query | Results (n) |
| --- | --- | --- |
| 1 | ‘malignant’ AND ‘ureteral’ AND ‘obstruction’ OR ‘malignant’ AND ‘ureteric’ AND ‘obstruction’ OR 'ureter obstruction'/exp AND 'neoplasm'/exp | 4,252 |
| 2 | ‘metal’ AND ‘stent’ OR ‘metallic’ AND ‘stent’ OR ‘metallic’ AND ‘ureteral’ AND ‘stent’ OR ‘covered’ AND ‘metal’ AND ‘stent’ OR ‘covered’ AND ‘metallic’ AND ‘stent’ OR 'metal'/exp OR 'metal stent'/exp | 2,063,520 |
| 3 | ‘polymer’ AND ‘stent’ OR ‘polymer’ AND ‘ureteral’ AND ‘stent’ OR 'double j' AND ‘stent’ OR ‘double’ AND ‘j’ AND ‘stent’ OR ‘polyurethane’ AND ‘stent’ OR 'polymer'/exp OR 'ureter stent'/exp | 1,015,999 |
| 4 | #1 AND #2 AND #3 | 230 |

3) Cochrane

| No. | Query | Results (n) |
| --- | --- | --- |
| 1 | “malignant ureteral obstruction” OR “malignant ureteric obstruction” | 27 |
| 2 | MeSH descriptor: [ureteral obstruction] explode all trees | 149 |
| 3 | MeSH descriptor: [neoplasms] explode all trees | 128,820 |
| 4 | #2 AND #3 | 11 |
| 5 | #1 OR #4 | 35 |
| 6 | “metal stent” OR “metallic stent” OR “metallic ureteral stent” OR “covered metal stent” OR “Covered metallic stent” | 2,792 |
| 7 | MeSH descriptor: [metals] explode all trees | 23,861 |
| 8 | #6 OR #7 | 26,273 |
| 9 | “polymer stent” OR “polymer ureteral stent” OR “double-J stent” OR “double J stent” OR “polyurethane stent” | 2,359 |
| 10 | MeSH descriptor: [polymers] explode all trees | 26,077 |
| 11 | #9 OR #10 | 28,060 |
| 12 | #5 AND #8 AND #11 | 5 |

4) Web of Science

| No. | Query | Results (n) |
| --- | --- | --- |
| 1 | "ALL=(“malignant ureteral obstruction” OR “malignant ureteric obstruction”) | 561 |
| 2 | "ALL=(“metal stent” OR “metallic stent” OR “metallic ureteral stent” OR “covered metal stent” OR “covered metallic stent”) | 19,469 |
| 3 | "ALL=(“polymer stent” OR “polymer ureteral stent” OR “double-J stent” OR “double J stent” OR “polyurethane stent” ) | 8691 |
| 4 | "#1 AND #2 AND #3 | 51 |

4) Scopus

| No. | Query | Results (n) |
| --- | --- | --- |
| 1 | “malignant AND ureteral AND obstruction” OR “malignant AND ureteric AND obstruction” | 5,216 |
| 2 | “metal AND stent” OR “metallic AND stent” OR “metallic AND ureteral AND stent” OR “covered AND metal AND stent” OR “covered AND metallic AND stent” | 88,459 |
| 3 | “polymer AND stent” OR “polymer AND ureteral AND stent” OR “double-j AND stent” OR “double AND j AND stent” OR “polyurethane AND stent” | 98,216 |
| 4 | ALL ( “malignant AND ureteral AND obstruction " OR “ malignant AND ureteric AND obstruction” ) AND ALL (“metal AND stent” OR “metallic AND stent” OR “metallic AND ureteral AND stent” OR “covered AND metal AND stent” OR “covered AND metallic AND stent”) AND ALL (“polymer AND stent” OR “polymer AND ureteral AND stent” OR “double-j AND stent” OR “double AND j AND stent” OR “polyurethane AND stent”) | 586 |

**Supplementary Table 2. Risk of bias assessment of the included studies**

**A. Risk of bias assessment of randomized controlled trials using the RoB 2 tool**

| **First author/year** | \| **Randomization** \| \| --- \| | **Deviations** | **Missing data** | **Outcome measurement** | **Reporting** | **Overall** |
| --- | --- | --- | --- | --- | --- | --- | --- |
| Jong Woo Kim, 2018 | Some concerns | Low | Low | Low | Low | Some concerns |
| Deok Hyun Han, 2025 | Low | Low | Low | Low | Low | Low |

**B. Risk of bias assessment of non-randomized comparative studies using the ROBINS-I tool**

| **First author/year** | **Confounding** | **Selection** | **Classification** | **Deviations** | **Missing data** | **Measurement** | **Reporting** | **Overall** |
| --- | --- | --- | --- | --- | --- | --- | --- | --- |
| Yue Chen, 2019 | Serious | Moderate | Low | Low | Low | Moderate | Low | Serious |
| Jumpei Asakawa, 2018 | Moderate | Moderate | Low | Low | Low | Low | Low | Moderate |
| Mari Ohtaka, 2021 | Moderate | Moderate | Low | Low | Low | Low | Low | Moderate |
| Hwan Hoon Chung, 2014 | Moderate | Moderate | Low | Low | Low | Low | Low | Moderate |
| E.T. Kim, 2021 | Moderate | Moderate | Low | Low | Low | Low | Low | Moderate |
